# Comparative genomics of spike, envelope, and nucleocapsid protein of severe acute respiratory syndrome coronavirus 2

**DOI:** 10.4314/ahs.v23i3.45

**Published:** 2023-09

**Authors:** Sufyan Sohail Khan, Anwar Ullah

**Affiliations:** Department of Biosciences, COMSATS University Islamabad, Islamabad, Pakistan

**Keywords:** *SARS-CoV-2*, COVID-19, pandemic, comparative genomics, spike protein, envelope protein, nucleocapsid protein

## Abstract

**Background:**

Severe Acute Respiratory Syndrome Coronavirus 2 (SARS-CoV-2) upsurge sprang up in Wuhan, China, in late December 2019.

**Objectives:**

Due to the exceptionally high mutation frequency, comparative genomics of viruses isolated throughout time and in various geographical locations are crucial. To better understand how SARS-CoV-2 heterogeneity has changed around the globe, this research was conducted.

**Methods:**

Nucleotide and protein sequences of *SARS-CoV-2, SARS-CoV*, and bat *SARS-like CoV* were extracted from the NCBI Virus database. The Wuhan *SARS-CoV-2* variant was used as a reference. Molecular Evolutionary Genetics Study performed the phylogenetic analysis, while the Genome Detective Coronavirus Typing Tool performed the mutational analysis.

**Results:**

The evolutionary research has revealed that bats are the primary host for coronavirus evolution and the origin of the formation of *SARS-CoV* and *SARS-CoV-2*. Numerous mutations have been discovered in the spike, envelope, and nucleocapsid protein.

**Conclusions:**

The current research findings may have an implication that facilitates the development of prospective immunization candidates/small pharmacological compounds targeting COVID-19.

## Introduction

Today, the entire world is facing a new global public health crisis. In December 2019, an upsurge sprang up in Wuhan, China, where a multitudinous of patients were suffering from a bizarre agglomeration of respiratory afflictions 1. The International Committee on Taxonomy of Viruses (ICTV) cleped the virus as Severe Acute Respiratory Syndrome Coronavirus 2 *(SARS-CoV-2)* on February 11^th^, 2020, and the ailment effectuated by SARS-CoV-2 was cleped as COVID-19 by the World Health Organization (WHO) 2,3. The *SARS-CoV-2* virus, which first appeared in Wuhan, progressively propagated throughout China and to other developed and developing countries throughout the world, ultimately erupting into a global pandemic, as proclaimed by WHO on March 11^th^, 2020 2,4. The pandemic has inflicted a myriad of people. As stated by Worldometer, an evaluation of 201, 064, 392 coronavirus cases, 4, 271, 371 demises, and 181, 054, 513 recovered have been reported (accessed on August 5^th^, 2021) 5.

Among all other nations, Pakistan has also been inflicted by the Wuhan virus. On February 26th, 2020, the Ministry of Health, Government of Pakistan, promulgated the first case of COVID-19 in Karachi. Another case was announced by the Federal Ministry of Health in Islamabad on the same day 6. As of 5th August 2021, there were 105, 3660 confirmed cases of which 23, 635 have died, 952, 616 have recovered, and 4050 are critical. The SARS-CoV-2 has greatly affected the Sindh province and is positioned at number 01 in proportion to the quota of COVID cases (with 392, 433 cases as of 5th August 2021) 7. Presently, Pakistan is enduring a fourth wave of COVID-19 endangered by the delta variant (B.1.617.2) also avowed as the Indian variant 8. The newly discovered variants of COVID-19, that is, South Africa and Brazilian variants, have been identified in Karachi during the third wave of COVID-19 9. In another report, researchers from Karachi disclosed that 70% of COVID-19 infirmities throughout Pakistan were attributed to the UK variant 10. However, based on new findings and data, researchers from the National Institute of Health, Islamabad, have claimed that 50% of COVID-19 manifestations in Pakistan are now attributed to the Delta (Indian) variant 11.

Genomics and Proteomics of COVID-19 have been delineated in relevant studies. Genomic studies have imparted that the structure of *SARS-CoV-2* is made up of *single-strand ribonucleic acid (ssRNA)*, comprising of four structural proteins, namely, Spike protein (S), an Envelope protein (E), Membrane protein (M), and Nucleocapsid (N) protein. In line with evolutionary studies, it portrays that it exhibits 96.2% homogenous to bat coronaviruses and 79.5% (<50% sequence identity) with *SARS-CoV* and *MERS-CoV* 12,13. *SARS-CoV-2* utilizes a distinct protein known as S-protein to bind specifically to the Angiotensin-Converting Enzyme 2 *(ACE 2)* of the host cell 14. Asides from spike protein, SARS-CoV-2 also embodies additional structural proteins such as E-protein and N-protein. E-protein is crucial for viral genome packaging and the synthesis of ion channels (IC), which are vital for virus-host contact and are frequently associated with pathogenicity 15,16. In contrast, the N-protein serves a variety of roles in the *CoV* virus replication. For example, in *SARS-CoV*, the N-protein has been shown to adhere to viral RNA and assemble it into *ribonucleoprotein (RNP)* complexes. 17,18

Due to the extremely high mutation frequencies, *SARS-CoV-2* is naturally prone to mutations, ensuing in genomic diversity 19. Because of the virus's fast evolution, generating vaccines and therapeutics may be problematic; consequently, comparative genomics of viruses isolated throughout time and in different regional areas is essential. A comparative study of the genomes of distinct *SARS-CoV-2, SARS-CoV*, and bat *SARS-like CoV* strains isolated would enable the identification and evaluation of the variable and preserved genomic regions moreover, this scientific understanding can help create efficacious vaccines and molecular epidemiological tracking. Thus, this research was implemented to investigate the evolution of *SARS-CoV-2* heterogeneity in other countries worldwide afflicted with COVID-19.

## Methods

### Ethical Approval

Ethical approval was attained by the Dean, Faculty of Science of COMSATS University Islamabad, Main Campus, Islamabad, Pakistan. This comparative genomic research was effectuated in August 2021 at the Department of Biosciences. This research entails no patient or animal models and was implemented by computational tools/software.

### Sequence Retrieval

Nucleotide sequences of *SARS-CoV-2* from Pakistan (Accession # MT240479.1), India (Accession # MZ558086.1), UK (Accession # MZ376737.1), South Africa (Accession # MZ202314.1), and Brazil (Accession # MZ397163.1) were retrieved from the NCBI Virus database. Likewise, the nucleotide sequences of *SARS-CoV* (Accession # NC_004718.3), and bat *SARS-like CoV* (Accession # MG772934.1) were also retrieved from the NCBI Virus database (https://www.ncbi.nlm.nih.gov/labs/virus/vssi/#/virus?SeqType_s=Nucleotide&VirusLineage_ss=SARS-CoV-2,%20taxid:2697049).Correspondingly, in synchrony to nucleotide sequences, protein sequences of S, E, and N-proteins of *SARS-CoV-2* variants, *SARS-CoV*, and bat *SARS-like CoV* were also retrieved from the NCBI Virus database. *SARS-CoV-2* from Wuhan, China, was used as a reference (Accession # NC_045512.2).

### Phylogenetic Analysis

Molecular evolutionary genetics analysis (MEGA) 11 version 11.0.10 software was exploited to generate and visualize the phylogenetic tree. A maximum likelihood approach with a bootstrap of 1000 replicates was employed for determining the best interfacing tree. The substitution model was computed to find the best DNA substitution model for the phylogenetic analysis.

### Mutational Analysis and Impact on Protein Stability

Genome detective Coronavirus typing tool (Version 1.17), which enables rapid identification and depiction of novel COVID genomes, executed mutational analysis. This tool allows for the input of up to 2000 sequences and completes the probe within seconds. This typing tool has been approved for classifying novel *SARS-CoV-2* among COVID species. We employed this typing method to detect mutation in the genome of *SARS-CoV-2* variants S, E, and N in comparison to Wuhan (China) *SARS-CoV-2*. Finally, we utilized an in-house software built in Perl and Python, MUpro server to forecast the impact of mutation on protein stability on *SARS-CoV-2* S, E, and N-protein.

## Results

### Evolutionary analysis

A phylogenetic probe was performed to determine variation among the genomes of *SARS-CoV-2, SARS-CoV*, and bat *SARS-like CoV*. MEGA 11 version 11.0.10 software was exploited for generating a phylogenetic tree. A maximum likelihood statistical method with a bootstrap of 1000 replicates was utilized to discern the best interfacing tree. The substitution model was computed, and the General Time Reversible + proportion of Invariant sites (GTR+I) model was found to be the best substitution model for the evolutionary analysis.

**Figure 1:**
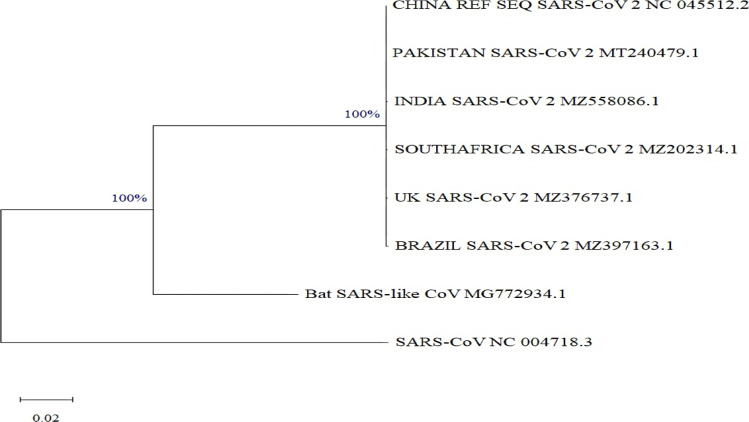
Phylogenetic Tree displaying an evolutionary variation of *SARS-CoV-2, SARS-CoV*, and bat *SARS-like CoV* complete (nucleotide) genome. The evolutionary history was inferred by using the Maximum Likelihood method and General Time Reversible model 57. The tree with the highest log likelihood (-72607.67) is shown. The percentage of trees in which the associated taxa clustered together is shown next to the branches. The rate variation model allowed for some sites to be evolutionarily invariable [+I], 46.45% sites). The tree is drawn to scale, with branch lengths measured in the number of substitutions per site. All positions with less than 95% site coverage were eliminated, i.e., fewer than 5% alignment gaps, missing data, and ambiguous bases were allowed at any position (partial deletion option). Evolutionary analyses were conducted in MEGA11 58

Our evolutionary research is evident that all the *SARS-CoV-2* variants form a clade that is all closely related to each other and to bat *SARS-like CoV*, which successively is related to *SARS-CoV*. This analysis reveals that only bat *SARS-like CoV* has a very close evolutionary relationship with *SARS-CoV-2* encountering an independent bifurcation from bat *SARS-like CoV*. The branch length of *SARS-CoV* portrays that it has diverged very early from bat SARS-like CoV. This evolutionary analysis strongly concurs with the fact that bats are the primary host for coronavirus evolution (Figure 1). The bootstrap values (100%) as depicted in Figure 1 robustly support this analysis.

### Comparative Genomics

**Figure 2:**
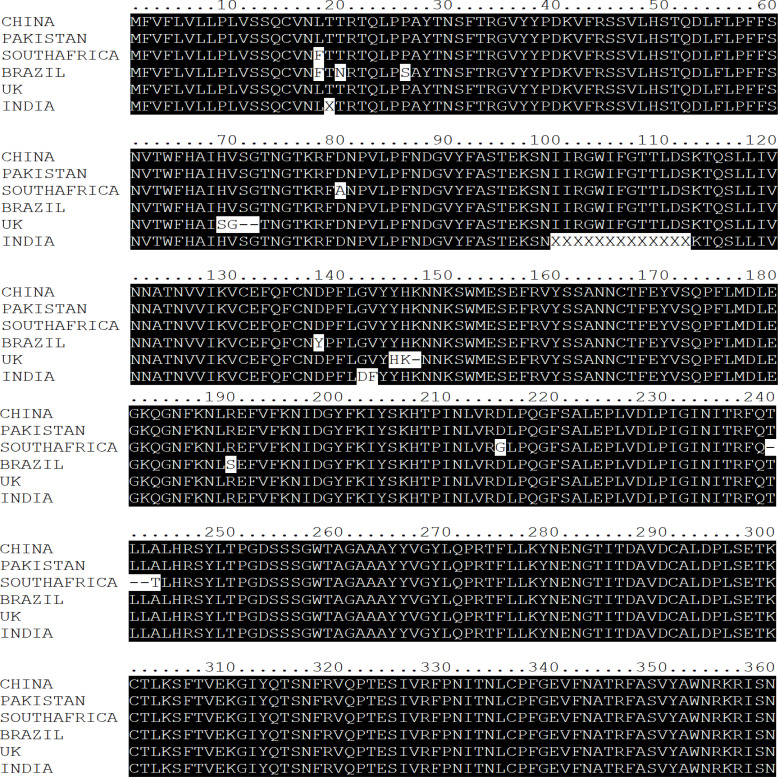

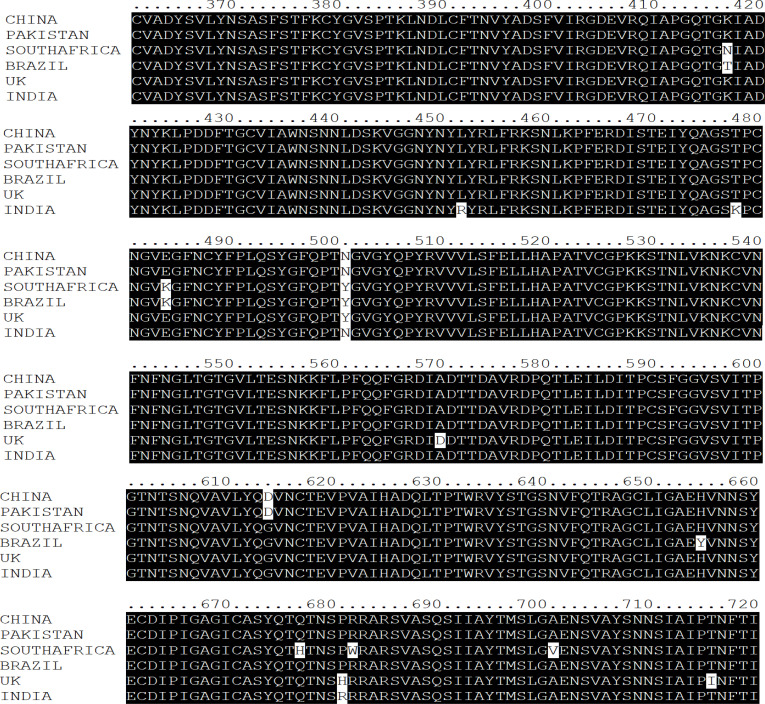

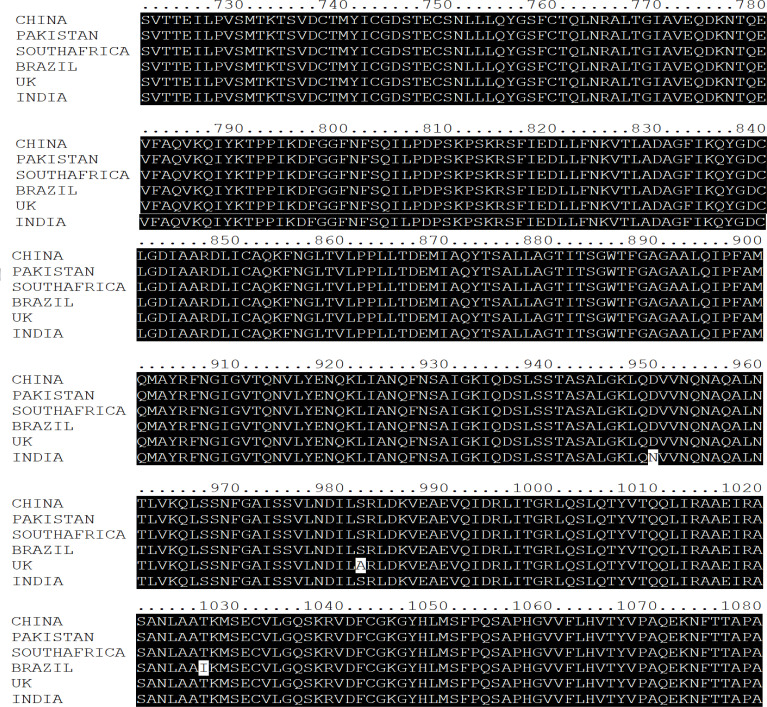

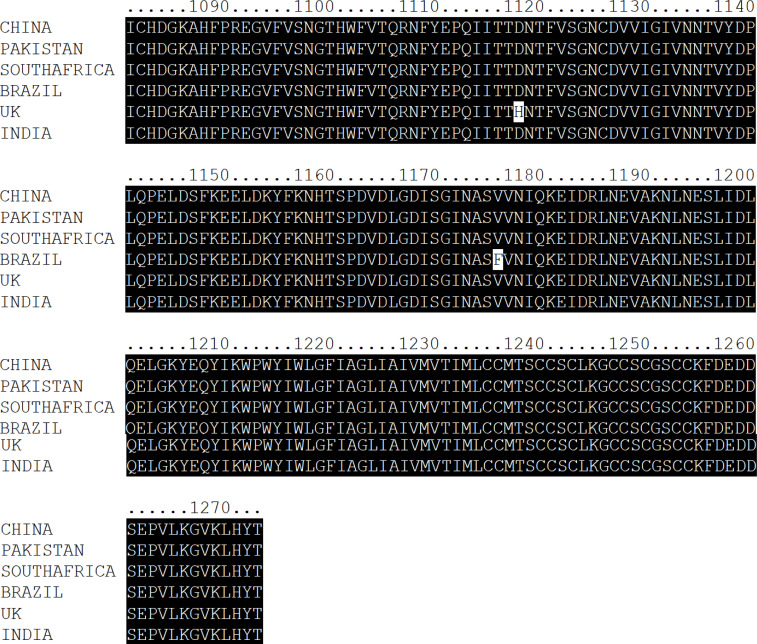
Multiple Sequence Alignment of SARS-CoV-2, SARS-CoV, and bat SARS-like CoV Spike Protein

**Figure 3:**
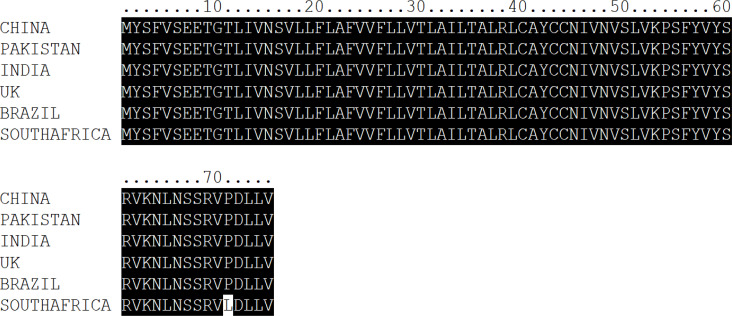
Multiple Sequence Alignment of SARS-CoV-2, SARS-CoV, and bat SARS-like CoV Envelope Protein

**Figure 4:**
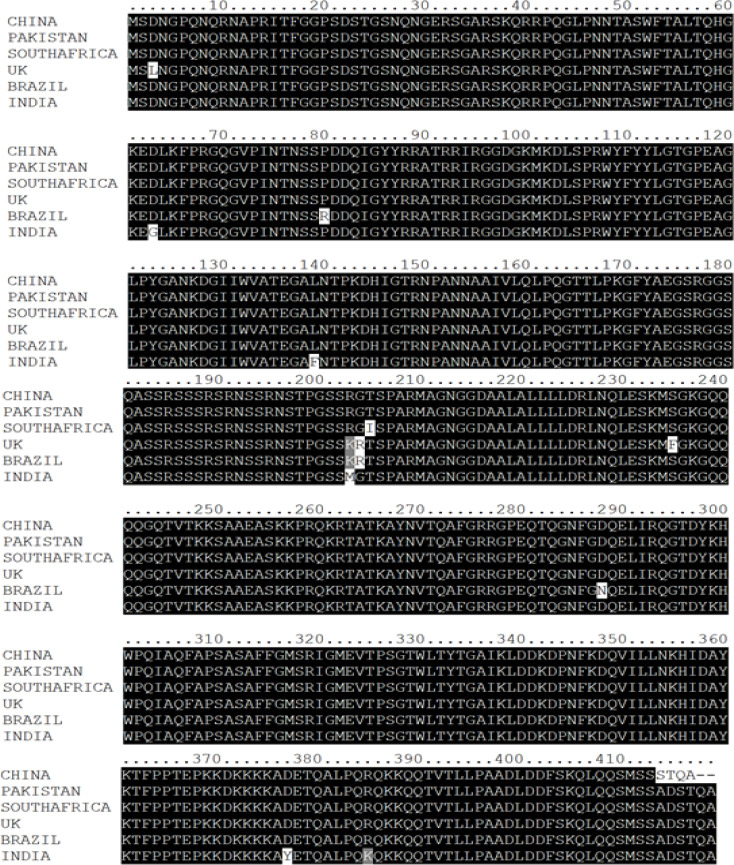
Multiple Sequence Alignment of SARS-CoV-2, SARS-CoV, and bat SARS-like CoV Nucleocapsid Protein

Multalin online tool 20 and Nucleotide BLAST (BLASTN) were used for comparative genomics, employing the *SARS-CoV-2* variant from Wuhan as a reference (Figure 2-4). The results of BLASTN have disclosed that all the SARS-CoV-2 S, E, and N-genomes exhibit 99% homogenous with Wuhan (China) *SARS-CoV-2* excluding the E-genomes of *SARS-CoV-2* (Pakistan, UK, Brazil, and India) and N-genome of *SARS-CoV-2* (Pakistan) which displays 100% similarity. In addition, *SARS-CoV* S, E, and N-genome display's a 78%, 94%, and 89% analogous with Wuhan (China) *SARS-CoV-2* whereas bat *SARS-like CoV* S, E, and N-genome displays 83%, 99%, and 91% homogenous (Supplementary Table 1-3). These results also authenticate the evolutionary variation among the genomes of *SARS-CoV-2, SARS-CoV*, and bat *SARS-like CoV* as delineated in Figure 1.

### Mutational Analysis

Mutational analysis was implemented by Genome Detective Coronavirus Typing Tool (Version 1.17) and validated by Multalin online tool 20. This tool has unearthed mutations eventuating in S, E, and Nprotein of *SARS-CoV-*2 variants. We found out that *SARS-CoV*-2 (India) has undergone 7 mutations, *SARS- CoV*-2 (UK) has undergone 9 mutations, *SARS-CoV*-2 (South Africa) has undergone 11 mutations, and *SARS- CoV*-2 (Brazil) has undergone 12 mutations, respectively. We have not witnessed any mutations in the S-protein of *SARS-CoV*-2 (Pakistan) (Table 1, Figure 2).

**Table 1:** Mutation in Spike protein of SARS-CoV-2 strains from diverse geographical regions

Protein	Origin	No. of Mutation	Mutation
**S**	Pakistan	0	-
	India	7	G142D (21987G>A)
			V143F (21989G>T)
			L452R (22917T>G)
			T478K (22995C>A)
			D614G (23403A>G)
			P681R (23604C>G)
			D950N (24410G>A)
	UK	9	H69_V70del (21766_21771delACATGT)
			Y144del (21992_21994delTAT)
			N501Y (23063A>T)
			A570D (23271C>A)
			D614G (23403A>G)
			P681H (23604C>A)
			T716I (23709C>T)
			S982A (24506T>G)
			D1118H (24914G>C)
	South Africa	11	L18F (21614C>T)
			D80A (21801A>C)
			D215G (22206A>G)
			L242_L244del (22286_22294delCTTGCTTTA)
			K417N (22813G>T)
			E484K (23012G>A)
			N501Y (23063A>T)
			D614G (23403A>G)
			Q677H (23593G>T)
			R682W (23606C>T)
			A701V (23664C>T)
	Brazil	12	L18F (21614C>T)
			T20N (21621C>A)
			P26S (21638C>T)
			D138Y (21974G>T)
			R190S (22132G>T)
			K417T (22812A>C)
			E484K (23012G>A)
			N501Y (23063A>T)
			D614G (23403A>G)
			H655Y (23525C>T)
			T1027I (24642C>T)
			V1176F (25088G>T)

Further, we have also observed one amino acid mutation of Proline (P) to Leucine (L) at the 71^st^ locus in the E-protein of *SARS-CoV-2* (South Africa). However, we have not witnessed any mutation in the E-protein of *SARS-CoV-2* (Pakistan, India, UK, and Brazil) as well in the N-protein of *SARS-CoV-2* (Pakistan) but have noted several mutations in the N-protein of *SARS-CoV-2* (India, UK, South Africa, and Brazil) (Table 2).

**Table 2:** Mutation in Envelope and Nucleocapsid protein of SARS-CoV-2 strains from various geographical locations

Protein	Origin	No. of Mutation	Mutation
**E**	South Africa	1	P71L (26456C>T)
	Pakistan, India, UK, and Brazil	0	-
**N**	Pakistan	0	-
	India	5	D63G (288461A>G)
			L139F (28690G>T)
			R203M (28881G>T)
			D377Y (29402G>T)
			R385K (29427G>A)
	UK	4	D3L (28280G>C 28281A>T 28282T>A)
			R203K (28881G>A 28882G>A)
			G204R (28883G>C)
			S235F (28977C>T)
	South Africa	1	T205I (28887C>T)
	Brazil	4	P80R (28512C>G)
			R203K (28881G>A 28882G>A)
			G204R (28883G>C)
			D288N (29135G>A)

### Impact on Protein Stability

We have distinguished the repercussion of these mutations on the protein stability by the MUpro server. It seems that the consequence of protein mutation diminishes the stability of the protein structure as demonstrated as negative (-) ΔΔG (Table 3).

**Table 3:** Repercussion of Mutation on Protein Stability

Protein Name	Mutation	Origin	Stability Effect (MUpro)
**S**	G142D	India	Decrease Stability (ΔΔG = -1.6291959)
	V143F		Decrease Stability (ΔΔG = -1.0725209)
	L452R		Decrease Stability (ΔΔG = -0.4547574)
	T478K		Decrease Stability (ΔΔG = -0.3452853)
	D614G		Decrease Stability (ΔΔG = -0.93148242)
	P681R		Decrease Stability (ΔΔG = -1.390031)
	D950N		Decrease Stability (ΔΔG = -0.61717494)
	N501Y	UK	Decrease Stability (ΔΔG = -1.7152495)
	A570D		Decrease Stability (ΔΔG = -1.8979773)
	D614G		Decrease Stability (ΔΔG = -0.93148242)
	P681H		Decrease Stability (ΔΔG = -1.158464)
	T716I		Decrease Stability (ΔΔG = -1.7281241)
	S982A		Decrease Stability (ΔΔG = -1.2467257)
	D1118H		Decrease Stability (ΔΔG = -0.90371524)
	L18F	South Africa	Decrease Stability (ΔΔG = -0.61079093)
	D80A		Decrease Stability (ΔΔG = -0.85116553)
	D215G		Decrease Stability (ΔΔG = -0.88640755)
	K417N		Decrease Stability (ΔΔG = -1.3481028)
	E484K		Decrease Stability (ΔΔG = -0.0090422293)
	N501Y		Decrease Stability (ΔΔG = -1.7152495)
	D614G		Decrease Stability (ΔΔG = -0.93148242)
	Q677H		Decrease Stability (ΔΔG = -0.80644929)
	R682W		Decrease Stability (ΔΔG = -0.62478789)
	A701V		Decrease Stability (ΔΔG = -1.5740659)
	L18F	Brazil	Decrease Stability (ΔΔG = -0.61079093)
	T20N		Decrease Stability (ΔΔG = -1.1205078)
	P26S		Decrease Stability (ΔΔG = -0.39125189)
	D138Y		Decrease Stability (ΔΔG = -1.01235)
	R190S		Decrease Stability (ΔΔG = -0.76374131)
	K417T		Decrease Stability (ΔΔG = -1.2705224)
	E484K		Decrease Stability (ΔΔG = -0.0090422293)
	N501Y		Decrease Stability (ΔΔG = -1.7152495)
	D614G		Decrease Stability (ΔΔG = -0.93148242)
	H655Y		Decrease Stability (ΔΔG = -0.8723021)
	T1027I		Decrease Stability (ΔΔG = -2.6637486)
	V1176F		Decrease Stability (ΔΔG = -1.4982363)
**E**	P71L	South Africa	Decrease Stability (ΔΔG = -1.9631091)
**N**	D63G	India	Decrease Stability (ΔΔG = -0.44971754)
	L139F		Decrease Stability (ΔΔG = -0.61518665)
	R203M		Decrease Stability (ΔΔG = -1.3051696)
	D377Y		Decrease Stability (ΔΔG = -1.277213)
	R385K		Decrease Stability (ΔΔG = -0.44097048)
	D3L	UK	Decrease Stability (ΔΔG = -1.53012)
	R203K		Decrease Stability (ΔΔG = -0.73977168)
	G204R		Decrease Stability (ΔΔG = -1.7379677)
	S235F		Decrease Stability (ΔΔG = -1.8826398)
	T205I	South Africa	Decrease Stability (ΔΔG = -1.9524886)
	P80R	Brazil	Decrease Stability (ΔΔG = -1.6718409)
	R203K		Decrease Stability (ΔΔG = -0.73977168)
	G204R		Decrease Stability (ΔΔG = -1.7379677)
	D288N		Decrease Stability (ΔΔG = -0.39236791)

## Discussion

In this study, we have examined *SARS-CoV-2 genomes* from Pakistan, India, the UK, South Africa, and Brazil, *SARS-CoV*, and bat *SARS-like CoV* to Wuhan (China) *SARS-CoV-2*. Evolutionary research has revealed that all the *SARS-CoV-2* variants form a clade that is all closely related to each other and to bat *SARS-like CoV*, which successively is related to *SARS-CoV*. This analysis reveals that only bat *SARS-like CoV* has a very close evolutionary relationship with *SARS-CoV-2* encountering an independent bifurcation from bat *SARS-like CoV*. The branch length of *SARS-CoV* portrays that it has diverged very early from bat *SARS-like CoV*. This evolutionary analysis strongly concurs with the fact that bats are the primary host for coronavirus evolution and the genesis of *SARS-CoV* and *SARS-CoV-2*, prompting scientists worldwide to ponder bats as a natural reservoir. (Figure 1). Our evolutionary analysis also concurs with other prior studies 21–25 moreover, is authenticated by BLASTN.

Coronaviruses (CoVs) have the longest genomes (26.4 to 31.7 kb) of any well-known RNA virus 4,26–28. The enormous genome size makes it flexible in acclimatizing and manipulating genes 26. The frequency of recombination in RNA viruses is rather substantial, henceforth enhancing virulence and thus is responsible for the development of speciation 29. The high frequency of recombination within the viral genome at various locations is perhaps one of the causes whereby *SARS-CoV-2* is accountable for both the variation in deaths and medical manifestations 30. The viral genome of *SARS-CoV-2* encodes four prime structural proteins: the S-protein, the N-protein, the M-protein, and an E-protein, which are all critical for the production of a functionally mature virion 31–37.

The *S-protein RBD* is the domain that precisely combines with ACE 2 to induce viral ingress into the host cell *38–42*. An assessment of the polypeptides of the S-protein of five *SARS-CoV-2* variants discovered polymorphisms in India, the UK, South Africa, and Brazil except for Pakistan at numerous nucleotide and amino acid positions (Table 1). The prognostication of protein stability employing theoretical or experimental techniques has been a significant topic of research for some years 43. Previous research suggests that a single point mutation at *RBD* is responsible for altering the epitope organization and, hence impairing *RBD* binding to *ACE* 2 44,45. A modification in this area of the S-protein may impair *RBD* adherence towards its receptors, consequently impacting viral penetration into the host genome.

E-protein is crucial for viral genome packaging and the synthesis of ion channels (IC), which are vital for virus-host contact and are frequently associated with pathogenicity 15,31,46. We have observed one amino acid mutation of Proline (P) to Leucine (L) at the 71^st^ locus in the E-protein of *SARS-CoV-2* (South Africa). We have not witnessed any mutation in the E-protein of *SARS-CoV-2* (Pakistan, India, UK, and Brazil) (Table 2). E-proteins are polypeptides with approximately 100 residues that are miniature components of virions but are extensively synthesized within infected cells 47,48. They exhibit a small hydrophilic N-terminus, one or more putative terminal transmembrane (TM) domains, and a less hydrophobic C-terminal tail (15). Previously, it was demonstrated that SNPs within the TMD domain of the E-protein impaired IC function and resulted in reduced viral virulence 49. Henceforth, E is a viable antiviral therapeutic target and immunization candidate against ^SARS-CoV-2^.

The N-protein serves a variety of roles in the *CoV* virus replication 17,18. For example, in *SARS-CoV*, the N-protein has been shown to adhere to viral *RNA* and assemble it into *RNP* complexes. The packed *RNPs* particles are found on the viral membrane's internal face, generating a distinct layer from the envelope proteins M, E, and S. Moreover, the association between N and the C-terminus of the M-protein may facilitate *RNP* localization 50,51. Numerous mutations at the nucleotide and amino acid positions in the N-protein of *SARS-CoV-2* have been discerned (Table 3). Prior studies have shown that Carboxyl-Terminal Domain (CTD) is essential for oligomerization 52.

It was also discovered that the *S-protein* was the most mutated of all the structural proteins investigated in this study. Among these variants, the most prominent are D614G, N501Y, E484K, K417N, K417T, and L452R. L452R mutation was discovered in the Indian *SARS-CoV-2 variant*, also referred to as *delta variant* (B.1.617.2), whereas conjunction of D614G and N501Y mutations was discovered in the UK *SARS-CoV-2* variant, also recognized as alpha variant (B.1.1.7). In South African *SARS-CoV-2 variants*, a combination of E484K, K417N, N501Y, and D614G was seen, widely known as beta variant (B.1.351), while a blend of K417T, E484K, N501Y, and D614G was discerned in the Brazilian *SARS-CoV-2* variant, commonly known as the gamma variant (P.1) 53–55. Our results concur with CDC 56. It is envisaged that the significant number of mutations found in structural proteins, particularly S-protein, will have an impact on the development of a vaccine/inhibitor against COVID-19.

To sum up, nucleotide and protein sequences of *SARS-CoV-2* from Pakistan, India, the UK, South Africa, and Brazil, *SARS-CoV*, and bat *SARS-like CoV* were evaluated and compared with Wuhan (China) *SARS-CoV-2*. Investigators uncovered variants in structural proteins that were unique to each nation (S-protein, E-protein, and N-protein). Furthermore, the MUpro server investigation indicated that mutations impair protein stability and impede inhibitor adhesion. The current research findings might facilitate the development of prospective immunization candidates/small pharmacological compounds targeting COVID-19.
